# Clinical characteristics of Graves’ disease following COVID-19 infection or vaccination: a multicenter case-control study

**DOI:** 10.55730/1300-0144.6096

**Published:** 2025-11-11

**Authors:** Asena GÖKÇAY CANPOLAT, Kemal AĞBAHT, Atilla Halil ELHAN, Mustafa CESUR, Ziynet ALPHAN ÜÇ, Seçkin AKÇAY, Hülya ILIKSU GÖZÜ, Mehmet AŞIK, Hayri BOSTAN, Bekir UÇAN, Tuğçe ŞAH ÜNAL, Merve YILMAZ, Ayşe KUBAT ÜZÜM, Mehmet Çağrı ÜNAL, Cüneyd ANIL, Ümmü MUTLU, Nurcan İNCE, Sevgül FAKI, Güven Barış CANSU, Mehmet Sercan ERTÜRK, Ayten OĞUZ, Mustafa AYDEMİR, Şefika Burçak POLAT, Oya TOPALOĞLU, Reyhan ERSOY, Bekir ÇAKIR, Ayşe Merve OK, Ersen KARAKILIÇ, Muhittin YALÇIN, Yusuf KAYHAN, Kader UĞUR, Dilek YAZICI, Alper GÜRLEK, Tülay OMMA, Emre Sedar SAYGILI, Adnan BATMAN, Banu KARA, Göknur YORULMAZ, Bahri EVREN, Füsun BALOŞ TÖRÜNER, Bülent Okan YILDIZ, Murat Faik ERDOĞAN, Faruk ALAGÖL, Erman ÇAKAL, Mustafa ŞAHİN

**Affiliations:** 1 Division of Endocrinology and Metabolic Diseases, Department of Internal Medicine, Faculty of Medicine, Ankara University, Ankara, Turkiye; 2 Clinic of Endocrinology and Metabolic Diseases, İskenderun Gelişim Hospital, Hatay, Turkiye; 3 Department of Biostatistics, Faculty of Medicine, Ankara University, Ankara, Turkiye; 4 Department of Endocrinology and Metabolic Diseases, Faculty of Medicine, Ankara Yüksek İhtisas University, Ankara, Turkiye; 5 Department of Endocrinology and Metabolic Diseases, Faculty of Medicine, Uşak University, Uşak, Turkiye; 6 Division of Endocrinology and Metabolic Diseases, Department of Internal Medicine, Faculty of Medicine, Marmara University, İstanbul, Turkiye; 7 Division of Endocrinology and Metabolic Diseases, Department of Internal Medicine, Faculty of Medicine, Haliç University, İstanbul, Turkiye; 8 Clinic of Endocrinology and Metabolic Diseases, Ankara Etlik City Hospital, Ankara, Turkiye; 9 Division of Endocrinology and Metabolic Diseases, Department of Internal Medicine, Faculty of Medicine, University of Health Sciences, Ankara Etlik City Hospital, Ankara, Turkiye; 10 Department of Internal Medicine, Samsun Gazi State Hospital, Samsun, Turkiye; 11 Division of Endocrinology and Metabolic Diseases, Department of Internal Medicine, Faculty of Medicine, Istanbul University, İstanbul, Turkiye; 12 Division of Endocrinology and Metabolic Diseases, Department of Internal Medicine, Faculty of Medicine, Dokuz Eylül University, İzmir, Turkiye; 13 Clinic of Endocrinology and Metabolic Diseases, Ankara Güven Hospital, Ankara, Turkiye; 14 Clinic of Endocrinology and Metabolic Diseases, Ankara Bilkent City Hospital, Ankara, Turkiye; 15 Department of Endocrinology and Metabolic Diseases, Faculty of Medicine, Kütahya Health Sciences University, Kütahya, Turkiye; 16 Division of Endocrinology and Metabolic Diseases, Department of Internal Medicine, Faculty of Medicine, İzmir Katip Çelebi University, İzmir, Turkiye; 17 Division of Endocrinology and Metabolic Diseases, Department of Internal Medicine, Faculty of Medicine, Kahramanmaraş Sütçü İmam University, Kahramanmaraş, Turkiye; 18 Division of Endocrinology and Metabolic Diseases, Department of Internal Medicine, Faculty of Medicine, Akdeniz University, Antalya, Turkiye; 19 Division of Endocrinology and Metabolic Diseases, Department of Internal Medicine, Faculty of Medicine, University of Health Sciences, Ankara Bilkent City Hospital, Ankara, Turkiye; 20 Division of Endocrinology and Metabolic Diseases, Department of Internal Medicine, Faculty of Medicine, Çanakkale Onsekiz Mart University, Çanakkale, Turkiye; 21 Division of Endocrinology and Metabolic Diseases, Department of Internal Medicine, Faculty of Medicine, Gazi University, Ankara, Turkiye; 22 Division of Endocrinology and Metabolic Diseases, Department of Internal Medicine, Faculty of Medicine, Ondokuz Mayıs University, Samsun, Turkiye; 23 Division of Endocrinology and Metabolic Diseases, Department of Internal Medicine, Faculty of Medicine, Fırat University, Elazığ, Turkiye; 24 Division of Endocrinology and Metabolic Diseases, Department of Internal Medicine, Faculty of Medicine, Koç University, İstanbul, Turkiye; 25 Division of Endocrinology and Metabolic Diseases, Department of Internal Medicine, Faculty of Medicine, Hacettepe University, Ankara, Turkiye; 26 Division of Endocrinology and Metabolic Diseases, Department of Internal Medicine, Faculty of Medicine, Lokman Hekim University, Ankara, Turkiye; 27 Division of Endocrinology and Metabolic Diseases, Department of Internal Medicine, Faculty of Medicine, Acıbadem University, İstanbul, Turkiye; 28 Division of Endocrinology and Metabolic Diseases, Department of Internal Medicine, Faculty of Medicine, Eskişehir Osmangazi University, Eskişehir, Turkiye; 29 Division of Endocrinology and Metabolic Diseases, Department of Internal Medicine, Faculty of Medicine, İnönü University, Malatya, Turkiye

**Keywords:** Graves’ disease, Graves’ orbitopathy, COVID-19 infection, inactivated virus COVID-19 vaccination, mRNA COVID-19 vaccination

## Abstract

**Background/aim:**

To describe Graves’ Disease (GD) associated with COVID-19 infection (COVID) or its vaccines (VAC) and to compare the clinical presentations, laboratory parameters, and short-term clinical course of the disease among different etiology groups (COVID, VAC, and GD control).

**Materials and methods:**

Included in this multicenter matched case–control, retrospective cohort study were 239 patients with newly diagnosed (n = 196) or recurrent GD (n = 43) associated with COVID (n = 79) or VAC (n = 160). Each case was matched (1:1) with a control who had been diagnosed with GD prior to COVID.

**Results:**

The median age of the entire group was 42 years (female:male = 137:102). Both the COVID (4.6-fold) and VAC (4.1-fold) groups demonstrated higher TSH receptor antibody (TRAb) titers (p < 0.001) compared with the control group (3.5-fold), as well as a higher proportion of recurrent cases. At baseline, the COVID group had higher free triiodothyronine (fT3) levels than the other groups. Graves orbitopathy (GO) was observed in 60 patients (12.6%), with a higher frequency in classical GD (18.4%). At baseline, the variables associated with thyrotoxicosis severity (defined as fT3 levels) were younger age, higher thyroid gland volume (TGV), and etiology, with the COVID and, to a lesser extent, VAC groups presenting with higher fT3 levels. The variables associated with GO were higher TGV, TRAb titers, and smoking, while no association with etiology was identified.

**Conclusion:**

The clinical course was similar in all groups other than in some laboratory findings. Although the frequency of GO associated with COVID and VAC was lower, the proportion of cases with a Clinical Activity Score of ≥3 was higher compared to GD. This pattern suggests a potentially stronger immunologic trigger in these cases.

## 1. Introduction

The relationship between the thyroid gland and the SARS-CoV-2 virus is intricate. COVID-19 (COVID) infection is associated with various thyroid manifestations, such as thyrotoxicosis, hypothyroidism, subacute thyroiditis, and nonthyroidal illness syndrome [1–4].

A sequential association has been observed between GD, a prevalent autoimmune disease, and COVID, with an earlier study reporting that GD usually manifests 30–60 days following the onset of the disease [5]. The breakdown of self-tolerance to thyroid antigens is the driving force behind thyroid autoimmunity [6]. There have also been reported cases of GD and/or Graves’ orbitopathy (GO) directly associated with the administration of SARS-CoV-2 vaccines [7–10].

Recent multicenter and meta-analytic studies have expanded the understanding of post-COVID and postvaccination Graves’ disease (GD). A systematic review of 57 postvaccine GD cases conducted by Triantafyllidis et al. reported that onset typically occurred within 2–3 weeks of exposure and that most patients responded to standard antithyroid therapy [11]. In a further study, SARS-CoV-2 vaccines and infection were noted to act as immune triggers in predisposed individuals through mechanisms such as molecular mimicry and bystander activation, without altering the fundamental disease phenotype [7]. More recent multicenter data suggest a modest increase in the incidence of GD during the pandemic, with cases showing higher TRAb titers and greater biochemical activity [12]. Addressing this issue, the present multicenter Turkish study provides complementary evidence through its direct comparison of COVID-associated, vaccine-associated, and classical GD, aiming to clarify whether the triggering etiology influences clinical presentation, immune activity, or short-term outcomes.

The presentation and clinical course were similar to those of typical GD not associated with COVID [13]. In an earlier cross-sectional study reporting an increase in the prevalence of GD, among the 66 GD reported cases diagnosed in 2021, 42 (63.6%) had been vaccinated within the 90 days preceding symptom onset [12]. While the utility of vaccines in managing the COVID pandemic was undoubtedly favorable, their potential side effects on thyroid autoimmunity remain a topic of debate. The pandemic has prompted reflections on the management of endocrine disorders and raised concerns about the impact of infections and vaccines on autoimmune endocrine diseases, both during the pandemic and in current clinical practice. There is also a need to clarify whether GD associated with these etiologies requires a different approach to those in the established guidelines.

The relationship between viral agents, vaccines, and autoimmune diseases has been well-documented; however, the postpandemic period offers a unique opportunity to observe the effects of a single virus and the associated vaccines.

In the present study, we document GD cases associated with COVID or its vaccines, and compare the clinical presentations, laboratory parameters, and short-term clinical course of the disease in different etiology groups (COVID, COVID-19 vaccines [VAC], and the classical GD [control]). To the best of our knowledge, this is the most extensive case series to date evaluating GD in association with the viral cause of the pandemic or the vaccines aimed at managing it.

## 2. Materials and methods

The study was approved by the General Directorate of Health Services of the Turkish Ministry of Health (March 24, 2022), and the local Institutional Research Ethics Committee (AUTF KAEK NO:2022/236). These approvals certify that the study was conducted in accordance with the ethical guidelines and principles outlined in the Declaration of Helsinki.

### 2.1. Patient Selection

For this multicenter matched case-control cohort study, the databases of 25 tertiary care centers were searched for relevant GD and/or GO cases from between June 2020 and June 2022, during the peak period of the pandemic. Cases with recent onset or recurrence (previously treated, in remission without antithyroid medication for at least 6 months, but recently relapsed) were identified, and those with documented COVID and those who had received mRNA-based VAC or inactivated virus-based VAC within the last 3 months before the onset of GD and/or GO were included in the study. The diagnoses of GD were based on clinical and laboratory data [14]. A thyroid ultrasound (US) was performed for the assessment of parenchymal blood supply patterns and parenchymal echogenicity, and to estimate thyroid gland volume (TGV). The volume of the lobe was calculated using the formula V (mL) = π/6 x depth x width x length (cm) [14]. Thyroid volume was calculated as the sum of the volumes of both lobes. The thyroid echogenicity was classified as i) diffuse homogeneous, ii) mild, iii) moderate, and iv) severe hypoechogenicity [15].

Excluded from the study were pediatric patients, those with toxic nodule(s), pregnant and lactating women, and cases with subacute thyroiditis (including COVID-associated subacute thyroiditis).

GO was diagnosed and treated according to the current guidelines of the European Group on Graves’ Orbitopathy and the European Thyroid Association [16], and clinical activity was assessed according to standardized criteria [17]. Proptosis was measured using a Hertel exophthalmometer [18].

A historical cohort was created after identifying the COVID and VAC groups to serve as the control group. Each case (from the COVID and VAC groups) was matched (1:1) with a control based on age and sex who had been diagnosed with GD and/or GO before 2019.

All appropriate cases were identified and formed the control group. Inclusion in the study was dependent on the availability of baseline TSH, fT4, fT3, and TRAb measurements, as well as thyroid ultrasound, and repeat measurements of TSH, fT4, and fT3 at a 3-month follow-up.

### 2.2. Laboratory assessment

Patients with COVID and a GD diagnosis within 3 months of a positive polymerase chain reaction (PCR) test were included in the COVID group, while those diagnosed with GD within 3 months following vaccination for SARS-CoV-2 were included in the VAC group [4], with the dates and names of the vaccines recorded according to patient declaration, and confirmed from electronic medical records. The SARS-CoV-2 vaccines were categorized as mRNA-based VAC (Pfizer-BioNTech) or inactivated virus-based VAC (CoronaVac, Sinovac, TURKOVAC. Also collected were data on the time between the date of vaccination and the onset of hyperthyroidism and/or eye symptoms.

The patients’ age, sex, smoking status, significant comorbidities and preexisting autoimmune conditions, thyroid function test results, thyroid autoantibodies, serum C-reactive protein (CRP) levels, thyroid US findings, and thyroid scan and uptake results (if available) were recorded. The type and dose of antithyroid medication, as well as any other medications, were noted. Thyroid function tests and changes in TRAb titers during the first 3 months following GD treatment were also documented.

The tests were performed in 23 laboratories affiliated with the Turkish Ministry of Health, and the laboratory data were standardized. All participating centers were accredited laboratories adhering to national and ISO 15189 quality standards. Thyroid function tests and TRAb assays were performed using third-generation immunoassays (Roche Elecsys, Abbott Architect, or Siemens Centaur), all of which were calibrated through national external quality assurance programs. Analyte values were normalized to each laboratory’s reference intervals and expressed as multiples of the upper or lower limit of normal for the harmonization of data: 1) to mitigate the difference in reference intervals across assay platforms and the lack of consistency in the availability of intra-laboratory z-score variance data from all centers; and (2) to take advantage of the validation of ratio-to-limit normalization reported in previous multicenter endocrine studies, thereby minimizing inter-assay bias when pooling heterogeneous data. The units were standardized before merging, and results from centers falling outside the acceptable quality-control limits were excluded.

### 2.3. Statistical assessment

First, to standardize the units of the variables studied, the fT3 and fT4 units were converted to pmol/L. Since some laboratories use different assays and diagnostic methods for thyroid autoantibodies, the measured value of the specific laboratory measurement was converted into a multiple of that parameter based on the upper limit given in the package insert for that assay. For example, if TRAb was measured at 17.5 IU/L and the upper limit of the reference assay was 1.75 IU/L, the variable was considered a factor of 10 and analyzed accordingly. Descriptive statistics were summarized as counts and percentages for categorical variables and as medians (with interquartile ranges of the 25th and 75th percentiles) for others, as most continuous variables were not normally distributed. Chi-square or Fisher’s exact tests were used for the comparison of categorical variables among groups, as appropriate. A Mann–Whitney U test was used to compare the continuous variables of two groups when the data were ordinal or not normally distributed. For ordinal and continuous variables across the three groups, discrepancies were evaluated with a Kruskal–Wallis test, a nonparametric analysis of variance. When the p-value obtained from the Kruskal–Wallis test statistic was statistically significant, Dunn’s test was used to identify the group responsible for the difference. Bonferroni correction was applied for all possible multiple comparisons to control the Type I error rate.

Linear regression analyses were performed to identify possible factors associated with baseline GD severity (defined as serum fT3 measurements at disease onset) [19, 20]. Variables with a p-value of < 0.25 in univariate linear regression, along with all variables of known clinical importance, were selected as candidates for the multivariate model using a purposive selection method.

A binary logistic regression model was used to estimate the factors potentially determining the presence of GO, and a purposive selection method was used for variable selection in binary logistic regression. Receiver operating characteristic (ROC) curves were employed to evaluate the diagnostic performance of the variables of interest and to determine cutoff values based on the Youden Index. The area under the ROC curves was calculated using the method outlined by Hanley and McNeil [21]. Hosmer–Lemeshow’s goodness-of-fit for the models was also checked. A two-tailed p-value of <0.05 was considered significant.

## 3. Results

### 3.1. GD cases associated with COVID and VAC

A total of 239 patients met the inclusion criteria for the study. The median age of the cohort was 42 years (33–52 years), with a female predominance (n = 185, 77.4%). Of the total, 79 patients (33.1%) were assigned to the COVID group while the remaining 160 (66.9%) were assigned to the VAC group. Within the VAC group, 30 (12.6%) received inactivated virus-based vaccines and 130 (54.4%) received mRNA-based vaccines. Clinical presentation manifested within the first month following exposure to the respective etiology in 107 (44.7%) cases, while in the remaining 132 (55.3%) GD developed within 3 months. Of the total GD cases, 195 (81.6%) were newly diagnosed and the remaining 44 (18.4%) were recurrent. Overall, the prevalence of GO was 9.2% (n = 22) in the cohort, including both the COVID and VAC groups (Table 1). Only 12 (5.0%) patients in the two groups tested negative for TRAb, and the GD in this minority of cases was confirmed via thyroid scintigraphy with diffusely increased thyroid gland activity and radioactive iodine uptake. Among all COVID and VAC cases, 36.8% were active smokers, and 20 (8.4%) had additional autoimmune diseases (Supplementary Table 1). Coexisting autoimmune diseases were observed in all groups, most commonly Hashimoto’s thyroiditis (9 of 20 cases, 45%), followed by type 1 diabetes mellitus (2) and premature ovarian failure (2). Nonthyroidal autoimmune conditions such as Addison’s disease, Sjögren’s syndrome, gluten-sensitive enteropathy, pemphigus vulgaris, and pernicious anemia were also represented, particularly among the mRNA vaccine-associated GD cases.

### 3.2. Classical GD cases

The median age of the control group was 41 (32–51) years, with a female predominance (n = 191, 79.9%). Of these cases, 31.8% were active smokers, and 215 (90.0%) were newly diagnosed with GD, whereas 24 (10%) were recurrent cases. The prevalence of GO was 18.4% (n = 38) in this cohort (Table 1), and only 19 (7.9%) patients in this group tested negative for TRAb.

### 3.3. Comparison of etiology groups

Age at presentation, sex, and smoking habits were comparable among the study groups (Table 1). Frequencies of concomitant autoimmune disease did not differ between the COVID and VAC groups. At baseline, serum fT4 levels, Tg Ab, TRAb positivity frequencies, and TGV were comparable among the three groups (Table 1). TRAb and TSH levels were both lower, and the neutrophil-to-lymphocyte ratio (NLR) and TPO Ab positivity frequency were higher in the control group than in the case groups. Serum fT3 levels were higher in the COVID group compared to the other two groups.

Laboratory data at 3-month follow-up are presented in Table 2. There was no difference among the three groups in terms of serum TSH, fT4, and TRAb levels, or in the change in serum TSH and fT4 levels at the 3-month follow-up. Methimazole (MMI) was the first-line antithyroid drug in all etiologic groups, administered to 401 of 478 patients. The three groups required similar antithyroid drug doses (10 mg/day MMI) at 3-month follow-up. The initial dosing and timing of therapy initiation were comparable between the COVID- and vaccine-associated GD groups. Treatment was typically started within the first week following diagnosis, with a median initial methimazole dose of 15 mg/day (range 10–20 mg/day), adjusted according to baseline fT4 and fT3 levels. No statistically significant difference was observed between the groups in either the starting dose or the time interval from symptom onset to treatment initiation (p > 0.05). Although the prescribed antithyroid drugs led to a more significant decrease in fT3 levels in the COVID group, they remained higher in this group. At -3-month follow-up, the control group had lower fT3 levels than the other groups.

Table 3 provides detailed information on the clinical presentation, eye examination results, and laboratory measurements of the GO cases. At the time of presentation, most of the clinical, laboratory, and radiographic features were comparable among the etiology groups, while lower TSH levels were recorded in the control group. Furthermore, Hertel measurements were higher, and the frequencies of GO and severe clinical activity (CAS ≥ 3) were greater in the COVID and VAC groups.

A linear regression analysis (dependent variable: serum fT3 level; independent variables: smoking habit, sex, age, TGV, TRAb titers, etiology) to identify predictors of thyrotoxicosis severity revealed the variables associated with baseline serum fT3 levels to be younger age, higher TGV, and etiology (Table 4). At presentation, COVID and VAC etiologies were associated with higher serum fT3 levels.

In a logistic regression analysis performed to identify predictors of GO, after adjusting for age and sex, the variables associated with GO were identified as TGV, TRAb titers at baseline, and smoking habits, but not the etiology. A Hosmer–Lemeshow test demonstrated a good model fit (χ^2^ = 6.85, df = 8, p = 0.553), indicating that the predicted probabilities were consistent with the observed outcomes (Table 5). The cutoff values used in the logistic regression analysis were derived from the ROC analysis, and the corresponding results are presented in Table 6.

## 4. Discussion

There is emerging evidence that both SARS-CoV-2 infection and vaccination can precipitate or reactivate Graves’ disease (GD) in those who are genetically susceptible through multiple, interrelated immunological mechanisms. Thyroid follicular cells express high levels of angiotensin-converting enzyme-2 (ACE2) and transmembrane protease serine 2 (TMPRSS2), facilitating viral entry and direct cytopathic injury, which may release thyroid antigens and promote epitope spread [22]. Molecular mimicry has been demonstrated between SARS-CoV-2 spike, nucleocapsid, and membrane proteins and thyroid autoantigens such as thyroperoxidase (TPO), thyroglobulin (Tg), and the TSH receptor, potentially inducing cross-reactive B- and T-cell responses [23]. The cytokine milieu during COVID – characterized by elevations in IL-6, TNF-α, and IL-17 – creates a Th1/Th17-skewed environment similar to that noted in GD, favoring bystander activation of autoreactive lymphocytes and germinal-center expansion with production of high-affinity, class-switched thyrotropin receptor antibodies [24, 25]. Excessive formation of neutrophil extracellular traps (NETs) during infection further exposes intracellular autoantigens and amplifies local inflammation [23]. In the vaccination setting, adjuvant-mediated innate immune stimulation – described within the framework of autoimmune/inflammatory syndrome induced by adjuvants (ASIA) – and the potent type I interferon and Th1-biased responses elicited by mRNA platforms may similarly breach self-tolerance and reactivate latent thyroid autoimmunity [24]. Collectively, these mechanisms – antigenic mimicry, bystander activation, epitope spread, NETosis, and adjuvant-driven innate activation – operate on a background of genetic susceptibility, particularly HLA-DRB1*03 and CTLA-4 variants, to initiate or unmask GD [25]. Although the absolute incidence of GD following SARS-CoV-2 infection or vaccination remains low, there is evidence that these immune challenges act as triggers rather than primary causes, unveiling autoimmune thyroid disease in predisposed hosts.

In the present study of new and recurrent GD and GO cases detected during the COVID-19 pandemic, the clinical presentations are compared within an age- and sex-matched cohort diagnosed with classical GD prior to the pandemic. Clinical presentations and even risk factors (presence of another autoimmune disorder, smoking, sex) were found to be comparable among the etiology groups. The COVID group presented with higher serum fT3 levels that remained higher at 3-month follow-up, despite the more significant decrease in the levels of this hormone. This was irrespective of serum fT4 levels, which were comparable among the groups at both baseline and at 3-month follow-up. Our results suggest that thyroid gland volume, younger age, and the attributable etiology (COVID and VAC) are independently associated with serum fT3 at GD presentation. The classical GD cohort had a higher frequency of GO, although a regression analysis yielded no independent association between the etiology and presence of GO. Instead, greater thyroid gland volume, higher TRAb titers, and smoking were independently associated with orbitopathy. When present, GO cases in the COVID and VAC groups were more frequently associated with severe clinical activity (CAS ≥ 3), possibly reflecting a more pronounced immune response. This may be related to the significantly higher TRAb titers observed in the COVID (4.6-fold) and VAC (4.1-fold) groups compared to the control group (3.5-fold). The higher proportion of cases with CAS ≥ 3 may reflect differences in immune activation intensity and autoantibody dynamics rather than simple prevalence variations. Both infection and vaccination elicit robust Th1/Th17-dominant immune responses and increased cytokine production (IL-6, IFN-γ, TNF-α), which may intensify orbital inflammation, despite similar TRAb positivity rates. Enhanced antigenic cross-reactivity between viral or spike-protein epitopes and orbital fibroblast antigens could also trigger more aggressive orbital inflammation in a subset of genetically susceptible patients. Alternatively, the apparent higher severity may partly reflect differences in the evaluation timing, as postvaccine or postinfection GD cases were detected early in their clinical course due to the heightened surveillance during the pandemic.

This study has clarified several important aspects of the clinical course of GD attributed to COVID and VAC. First, it was found that GD (regardless of the attributable etiology) occurs more frequently in women and at an age similar to that of classical GD, which aligns with established GD epidemiologic data [8, 11]. This supports the role of genetic and environmental factors, including the immunomodulatory effects of sex hormones, such as the reducing effect of estrogen on regulatory T-cell activity, in autoimmune thyroid diseases [5]. A report on vaccine-associated GD early in the COVID pandemic claimed that age at disease onset was higher, and the proportion of males was higher in cases of vaccine-associated GD [11]. A study by Di Filippo et al. assessed the early vaccination period covering the priority population at the highest risk for more severe COVID; however, the population was mainly unvaccinated at the time of the study, and so the cohort was primarily older adults and men with comorbidities [26]. The present study covers the entire active vaccination period.

It is notable that the prevalence of ophthalmopathy in the VAC and SARS-CoV-2 groups was ≤ 10%, which is considerably lower than that observed in a historical cohort (18.4%) and substantially below the 25%–50% rates reported in the literature [27]. Our regression analysis, however, revealed no independent association between the etiology and the presence of GO. Furthermore, our results suggest that the frequency of GO in COVID- or vaccine-associated GD is not increased, at least in the early course of the disease.

Most of the cases (81.6%) in the present study were newly diagnosed, while the remainder were recurrent GD cases. A study analyzing the outcomes of patients with GD 25 years after initiating antithyroid medication showed recurrence rates of 45% within two years of stopping antithyroid medication [28]. Although the frequency of recurrent cases in the COVID and VAC groups was slightly higher than in classical GD cases, this finding must be interpreted in the context of the inherently high recurrence rate as the natural course of Graves’ disease. Therefore, longitudinal studies are needed to better understand the recurrence patterns of COVID-associated GD and GO.

Most of the laboratory measurements were comparable among the three etiology groups. TRAb levels were higher at baseline, whereas NLR was lower in the COVID and VAC groups when compared to the control group. It is interesting to note that despite the similar fT4 levels and higher fT3 levels in the COVID group, TSH was suppressed to a lower level in the control group, than in the COVID and VAC groups. After receiving similar MMI doses, all three groups achieved similar TSH, fT4, and TRAb levels at 3-month follow-up, but despite the further decrease in fT3 levels in the COVID group, they remained higher in the COVID and VAC groups. The variables associated with thyrotoxicosis severity (baseline fT3 levels) were higher TGV, younger age, and COVID or VAC etiology, confirming the role of genetic predisposition, which accounts for 79% of the GD risk, and environmental factors, which account for 21% [29].

As approximately 70% of genes associated with autoimmune thyroid disorders are implicated in T-cell function [29], it was no surprise that approximately 10% of the study population had concomitant autoimmune disorders (Supplementary Table 1). It was noted in a recent article that, according to nationwide United States networks, severe COVID was linked to the highest risk of new autoimmune diseases, with adjusted hazard ratios ranging from 1.14 to 3.14. Among individuals with evidence of COVID, the most frequently observed autoimmune diseases were thyroid disorders, psoriasis or psoriatic arthritis, and inflammatory bowel disease [30]. Furthermore, in recent study conducted in Italy, a higher prevalence of positive thyroid autoantibody titers was revealed following the pandemic, particularly in women, when compared with prepandemic controls [31]. All these findings support the relationship between infection and autoimmune diseases.

The lower NLR noted in the COVID and VAC groups may also indicate the role of T-cell involvement in the pathogenesis and severity of GD, as evidenced by the higher fT3 levels at presentation in these groups. On the other hand, the less suppressed TSH levels in GD cases encountered during the COVID pandemic, despite the higher fT3 levels, may suggest a possible role for deiodinases and/or changes in the sensitivity of thyrotropic cells to circulating thyroid hormones.

There have been a few cases of GO associated with COVID or vaccines [9, 32]. Our regression analysis revealed an independent association of TGV, TRAb titers, and smoking, regardless of the underlying etiology. Thyroid volume has been reported to correlate with the severity of GO at initial observation, especially with exophthalmometry and CAS [33]. Possible explanations for these associations include the pathogenic role of antigens shared by the thyroid and orbital tissues, as well as the more reactive immune system encountered in patients with a large goiter, which may result in more severe thyroid and eye disease. In our study, although thyroid volume and TRAb positivity rates were comparable across all three groups, TRAb titers were higher in the COVID and VAC groups; nevertheless, the prevalence of GO was lower in these two groups.

On the other hand, the proportion of people with CAS ≥ 3 was higher in the COVID and VAC groups. Although the overall frequency was lower, the greater severity observed may be indicative of underlying complex immunological mechanisms. That said, the small sample size in our study cohort with GO precludes definitive conclusions, and more extensive study groups are required for confirmation.

Among the strengths of the present study are its analysis of the most extensive case series from the COVID-19 pandemic and its coverage of the entire pandemic period. The study sourced the most comprehensive COVID and postvaccination GD data, including cases from cities with different geographical and economic characteristics. Furthermore, the study centers were all tertiary care centers, and all those who contributed cases to the study were endocrinologists with expertise in thyroid US. The attributed etiology was clear, and there was no overlap among the etiology groups. The control group, formed out of matching cases, had been diagnosed before the pandemic, and the matching was based on sex, age, and the closest seasonal day. This approach was applied to avoid any selection bias.

There are, however, some limitations that should be recognized. First, the follow-up period was short, and long-term outcome data are unavailable. The relatively short follow-up may underestimate late relapses or delayed ophthalmic manifestations, which often occur beyond 6–12 months after diagnosis or treatment initiation. Second, the laboratory examination methods and assays varied across the study centers, although all the laboratories were certified, and the units were equalized within the reported laboratory data. Third, the definition of thyrotoxicosis severity in our study can be criticized. Free T3 may provide superior clinical insight to free T4, as it more directly reflects peripheral thyroid hormone activity, particularly in early or atypical presentations of hyperthyroidism and in certain nonthyroidal illnesses [20, 34, 35]. We selected serum fT3 as the primary surrogate of disease severity as it directly reflects the biologically active thyroid hormone and is the most sensitive indicator of peripheral thyroid hormone metabolism during acute and subacute systemic illness. Previous studies involving both COVID and non-COVID cohorts have consistently reported that low fT3 (low-T3 syndrome) correlates with illness severity, inflammatory burden, and unfavorable outcomes more robustly than fT4 or the fT4/fT3 ratio. Although the fT4/fT3 ratio may indirectly indicate altered deiodinase activity, its interpretation can be confounded by assay heterogeneity, concurrent glucocorticoid exposure, and delayed sampling after clinical stabilization. Furthermore, given the multicenter design and inter-assay variability in fT4 measurements, using the fT4/fT3 ratio could have led to the introduction of additional analytical noise. To maintain uniformity and to minimize assay-related bias, we therefore focused on fT3 alone as a physiologically meaningful and analytically stable marker. Finally, our study was unable to compare the GD findings with prepandemic trends. In the present study, all cases of GD detected during the pandemic were confirmed to be associated with COVID (tested with PCR) or its vaccines, although our study cannot comment on the actual trends of GD during that period, as some possible asymptomatic COVID cases may have been unintentionally excluded. On the other hand, the control group was carefully constructed to include GD cases diagnosed in the prepandemic period.

Our findings align with prior systematic reviews showing that COVID infection and vaccination can act as immunologic triggers for GD, but do not necessarily alter the overall disease spectrum. In a review of 57 postvaccine GD cases by Triantafyllidis et al., most cases presented within 15 days, were typically mild to moderate in severity, and responded well to standard antithyroid therapy [11]. Similarly, Caron et al. emphasized that vaccine-induced autoimmune thyroid disease generally mirrors the classical phenotype, suggesting a shared pathophysiology driven by molecular mimicry and bystander activation [7]. In contrast to these meta-analyses, our large, multicenter Turkish cohort (n = 478) provides population-level data showing comparable prevalence but higher biochemical and immunologic activity (elevated fT3 and TRAb titers) in COVID- and vaccine-associated GD, and a greater proportion of severe GO, despite the lower overall frequency. These results add to the wealth of prior case-based findings and underscore the importance of immune activation intensity rather than vaccine platforms alone in modulating disease expression. Clinically, these observations highlight the need for early ophthalmologic assessment in post-COVID or postvaccination GD, especially when TRAb titers are high or fT3 levels are disproportionately elevated. Endocrinologists should remain vigilant for early thyrotoxic or ocular symptoms following SARS-CoV-2 infection or vaccination, particularly in patients with preexisting autoimmune disease or a personal/family history of thyroid autoimmunity.

In conclusion, GD triggered by COVID or vaccination may manifest with more severe thyrotoxicosis and GO (although less frequent) compared to other causes, attributable to the enhanced immune response and potential systemic inflammation caused by the virus or the vaccines. It is important to note, however, that GD caused by COVID or its vaccines is manageable with the appropriate antithyroid medication. Although the most recent wave of SARS-CoV-2 has subsided, the risk of future viral pandemics remains a persistent concern. What is clear, however, is that vaccines targeting viral pathogens – such as those used to prevent cancers – will continue to play a critical role in public health. From an endocrinological standpoint, we recommend vigilance in those with preexisting autoimmunity, given the potential risk of vaccine- or infection-associated autoimmune manifestations.

## Supplementary Information



## Figures and Tables

**Table 1:** Baseline clinical and laboratory evaluation of GD/GO cases where the data was available.

	COVID (n = 79)	VAC (n = 160)	Control (n = 239)	p-value
Age (years), Median (P25–P75)	42 (31.0–47.0)	44 (33.0–53.8)	41 (32.0–51.0)	0.111
Sex, female, n (%)	62 (78.5)	123 (76.9)	191 (79.9)	0.767
Smoking habits a, n (%)	27/68 (39.7)	47/133 (35.3)	76/239 (31.8)	0.448
Presence of another autoimmune disease, n (%)	5 (6.3)	15 (9.4)		0.424
Clinical presentation within the first month following the exposure to etiology, n (%)	37 (46.8)	70 (43.8)		0.652
De novo GD cases, n (%)	65 (82.3)	130 (81.2)	215 (90.0)	*0.006*
Recurrent GD cases, n (%)	14 (17.7)	30 (18.8)	24 (10.0)	*0.043*
Presence of GO, n (%)	6 (7.6)	16 (10.0)	38 (18.4)	*0.016*
**Laboratory and radiological evaluations**				
TSH (basal) mıu/L, Median (P25–P75)	0.010 (0.005–0.010)	0.010 (0.005–0.010)	0.005 (0.001–0.010)*	*<0.001*
fT4 pmol/l, Median (P25–P75)	37.3 (26.4–51.5)	34.3 (23.2–50.2)	37.8 (26.6–52.3)	0.352
fT3 pmol/l, Median (P25–P75)	16.6 (11.7–26.8)#	13.2 (8.0–21.5)	12.0 (7.9–18.7)	0.001
fT4 measured/upper limit^a^, Median (P25–P75)	1.70 (1.20–2.34)	1.56 (1.05–2.28)	1.72 (1.21–2.38)	0.352
fT3 measured/upper limit^a^, Median (P25–P75)	2.44 (1.72–3.94)#	1.94 (1.18–3.16)	1.76 (1.16–2.75)	0.001
TRAb measured/upper limit^b^, Median (P25–P75)	4.6 (2.2–13.4)	4.1 (1.8–10.3)	3.5 (1.6–6.4)&	<0.001
TPO Ab positivity a, b, n (%)	39/65 (60.0)	90/137 (65.7)	145/190 (76.3)	*0.019*
Tg Ab positivity a, b, n (%)	32/59 (54.2)	62/122 (50.8)	92/172 (53.5)	0.873
TRAb positive cases, n (%)	63/69 (91.3)	141/146 (96.6)	220/239 (92.1)	0.167
CRP (0–5 mg/dL), Median (P25–P75)	2.0 (0.9–4.0)	1.5 (0.6–3.0)	1.9 (0.8–3.1)	0.206
Neutrophyle/lymphocyte ratio, Median (P25–P75)	1.5 (1.0–2.2)	1.7 (1.3–2.1)	1.9 (1.4–3.0)%	<0.001
Thyroid gland volume (mL), Median (P25–P75)	16.7 (12.0–22.0)	17.2 (13.3–25.1)	18.8 (14.2–25.0)	0.133

* Different from both COVID and VAC (p = 0.043 and p < 0.001, respectively);

# Different from VAC and Control (p = 0.046 and p = 0.001, respectively);

& Different from both COVID and VAC (p = 0.001 and p = 0.001, respectively);

% Different from both COVID and VAC (p < 0.001 and p = 0.003, respectively)

* Data was available for number of cases

** Independent Kruskal-Wallis Test and Chi-Square Test

a numbers are presented for available cases

b since there were differences for kits among the study centers, these data were standardized based on the upper limits of that laboratory’s reference kit

P25: 25^th^ percentile, P75: 75^th^ percentile, COVID: Covid-19 infection associated, VAC: Vaccination-associated, GD: Graves’ disease, GO: Graves’ orbitopathy, TSH: Thyroid stimulating hormone, fT3: Free tri-iodothyronine, fT4: Free thyroxine, TRAb: TSH receptor antibody, CRP: C reactive protein, AST: Aspartate aminotransferase, ALT: Alanine transaminase, GGT: Gamma-Glutamyl transferase ALP: Alkaline phosphatase, NLR: Neutrophil/lymphocyte ratio

**Table 2:** Clinical and laboratory characteristics of cases with COVID-associated, vaccine-associated, and control GD cases at 3-month follow-up.

	COVID (n = 79)*	VAC (n = 160)*	Control (n = 239)*	p-value**
**TSH smıu/L, Median (P25**–**P75)**	0.20 (0.01–1.93)	0.26 (0.04–1.84)	0.30 (0.01–2.38)	*0.758*
**fT4, pmol/L, Median (P25**–**P75)**	12.8 (10.3–17.0)	13.5 (11.5–17.8)	13.1 (10.4–18.1)	*0.357*
**fT3, pmol/L, Median (P25**–**P75)**	5.0 (4.3–6.4)	5.1 (4.4–6.0)	4.6 (3.5–5.9)#	*<0.001*
**fT4 measured/upper limit**^a^, **Median (P25**–**P75)**	0.58 (0.47–0.77)	0.61 (0.52–0.81)	0.60 (0.47–0.82)	*0.357*
**fT3 measured/upper limit**^a^, **Median (P25**–**P75)**	0.74 (0.63–0.93)	0.75 (0.65–0.88)	0.68 (0.51–0.86)#	*<0.001*
**TRAb measured/upper limit**^a^, **Median (P25**–**P75)**	1.6 (0.5–10.0)	1.8 (1.0–3.3)	1.6 (0.9–3.0)	*0.572*
**ΔTSH, mıu/L, Median (P25**–**P75)**	0.15 (0.001–1.78)	0.20 (0.01–1.72)	0.28 (0.01–2.28)	*0.871*
**ΔfT4, pmol/L, Median (P25**–**P75)**	−24.5 (−41.4– −13.1)	−19.0 (−38.1– −8.7)	−22.4 (−36.2– −12.0)	*0.274*
**ΔfT3, pmol/L, Median (P25**–**P75)**	−12.1 (−21.6– −6.2)$	−7.7 (−16.1– −2.7)	−6.7 (−13.0 – −3.3)&	*0.005*
**Δ% fT4, pmol/L, Median (P25**–**P75)**	−64.5 (−76.3– −44.3)	−56.3 (−72.7– −33.9)	−62.2 (−75.4– −42.2)	*0.160*
**Δ% fT3, pmol/L, Median (P25**–**P75)**	−70.8 (−77.3– −53.1)£	−59.6 (−75.4 – −30.7)	−59.4 (−74.6– −42.0)&	*0.036*
**Antithyroid (MMI) dosage, mg/day (n = 401), Median (P25**–**P75)**	10.0 (5.0–15.0)	10.0 (5.0–15.0)	10.0 (5.0–15.0)	*0.708*

P25: 25^th^ percentile, P75: 75^th^ percentile, COVID: COVID-19 infection associated, VAC: Vaccination associated, TSH: Thyroid stimulating hormone, fT3: Free tri-iodothyronine, fT4: Free thyroxine, TRAb: TSH receptor antibody, MMI: Methimazole Δ: The change of laboratory parameter from baseline,

* Data was available for number of cases, n: number of patients,

** Kruskal Wallis Variance Analysis and Chi-Square Test,

a since there were differences for kits among the study centers, these data were standardized based on the upper limits of that laboratory’s reference kit

# Different from VAC and COVID (p < 0.001 and p = 0.019, respectively);

$ Different from VAC and Control (p = 0.020 and p = 0.004, respectively);

£ Different from VAC (p = 0.032);

& Different from VAC and COVID (p = 0.039 and p = 0.044, respectively).

**Table 3:** Analysis of GO cases.

	COVID (n = 6)	VAC (n = 16)	Control (n= 38)	p-value
**Age (years), Median (P25**–**P75)**	43.0 (39.3–44.3)	41.5 (30.5–49.0)	40.0 (30.0–51.0)	0.834
**Sex, female**	5 (83.3)	10 (62.5)	30 (78.9)	0.392
**Smoking habits** a **(n = 57)**	3/6 (50.0)	5/13 (38.5)	21/38 (55.5)	0.578
**TSH (basal) mıu/L, Median (P25**–**P75)**	0.01 (0.01–0.03)	0.01 (0.01–0.05)	0.006 (0.001–0.010)#	<0.001
**Baseline fT4 pmol/L, Median (P25**–**P75)**	41.0 (36.6–58.0)	37.8 (27.4–51.0)	45.4 (29.5–61.8)	0.594
**Baseline fT3 pmol/L, Median (P25**–**P75)**	23.0 (16.1–27.1)	16.1 (11.4–27.9)	12.6 (7.1–20.5)	0.133
**fT4 measured/Upper Limit pmol/L, Median (P25**–**P75)**	1.9 (1.7–2.6)	1.7 (1.2–2.3)	2.2 (1.3–2.8)	0.594
**fT3 measured/Upper Limit pmol/L, Median (P25**–**P75)**	3.4 (2.4–4.0)	2.4 (1.7–4.1)	1.8 (1.0–3.0)	0.133
**Baseline TRAb measured/upper limit IU/L, Median (P25**–**P75)**^a^	12.5 (4.7–48.7)	5.7 (2.2–26.7)	5.4 (2.7–17.4)	0.491
**CAS ≥ 3**	3/6 (50)	8/15 (53.3)	4/30 (13.3)	0.006
**Thyroid gland volume, mL, Median (P25**–**P75)**	23.5 (6.3–NA)	22.7 (18.2–28.9)	20.7 (14.2–28.4)	0.642
**CRP (mg/dl), Median (P25**–**P75)**	8.0 (3.1–22.5)	2.2 (0.5–3.1)£	3.1 (1.8–4.1)	0.037
**Hertel measures Right eye (mm), Median (P25**–**P75)**	23.0 (20.0–25.0)	20.5 (20.0–22.0)	20.0 (18.0–21.0)&	0.025
**Hertel measures Left eye (mm), Median (P25**–**P75)**	21.0 (19.5–25.5)	22.0 (20.0–23.8)	19.0 (18.0–21.0)%	0.019

P25: 25^th^ percentile, P75: 75^th^ percentile, COVID: COVID-19 infection associated, VAC: Vaccination associated, TSH: Thyroid stimulating hormone, fT3: Free tri-iodothyronine, fT4: Free thyroxine, TRAb: TSH receptor antibody GO: Graves’ orbitopathy, CAS: Clinical activity score, CRP: C reactive protein

** Independent Kruskal Wallis Test and chi-square test

a numbers are presented for available cases

b since there were differences for kits among the study centers, these data were standardized based on the upper limits of that laboratory’s reference kit

# Different from VAC (p < 0.001);

£ Different from COVID (p = 0.030);

& Different from COVID (p = 0.032);

% Different from VAC (p = 0.043)

**Table 4:** Linear regression analysis of baseline serum fT3 determinants (n = 478).

	Univariate			Multivariable				
	B	Std. Error	p	B	Std. Error	95% CI for B	Beta	p
**Sex**	1.528	1.139	0.180					
**Age**	−0.104	0.037	0.004	−0.104	0.040	(−0.182– −0.026)	−0.136	**0.009**
**Smoking**	1.213	1.008	0.229					
**Thyroid gland volume (ml)**	0.163	0.035	**<0.001**	0.171	0.035	(0.103–0.239)	0.255	**<0.001**
**Baseline TRAb measured/upper limit**	0.033	0.021	0.115					
**Etiology1 (COVID vs. control)**	5.441	1.327	**<0.001**	4.110	1.541	(1.078–7.142)	0.144	**0.008**
**Etiology2 (VAC vs. control)**	1.666	1.023	0.104	2.909	1.086	(0.773–5.044)	0.143	**<0.001**

B: Regression coefficient; Beta: Standardized regression coefficient; CI: Confidence interval

R^2^ = 0.110; Adjusted R^2^ = 0.100

**Table 5:** Logistic regression analysis of presence of Graves’ orbitopathy (n = 60).

	Univariate			Multivariable		
	OR	95% CI for OR	p	OR*	95% CI for OR	p
**Smoking**	2.299	(1.305–4.051)	0.004	2.079	(1.068–4.047)	0.031
**Sex (Male vs. female)**	1.338	(0.709–2.525)	0.370			
**Age ≥ 41 years**	0.909	(0.527–1.568)	0.732			
**Basal fT3 ≥ 11.40 (pmol/L)**	1.915	(1.049–3.496)	0.034			
**Thyroid gland volume ≥ 17.90 (ml)**	2.481	(1.304–4.719)	0.006	2.256	(1.099–4.634)	0.027
**Baseline TRAb measured/Upper Limit ≥ 5.66**	2.676	(1.524–4.696)	<0.001	2.091	(1.078–4.057)	0.029
**Etiology1 (COVID vs. control)**	0.366	(0.148–0.902)	0.029			
**Etiology2 (VAC vs. control)**	0.494	(0.265–0.923)	0.027			
**Neutrophyle/lymphocyte ratio**	1.070	(0.842–1.359)	0.580			

OR: Odds ratio, CI: Confidence interval

Hosmer and Lemeshow Test Statistics = 6.850, df = 8, p = 0.553

* Adjusted for age and sex

**Table 6:** Results of the Receiver Operating Characteristics Analysis.

	AUC ± SE P value	95% CI	P	Cut-off points	Sensitivity	Specificity
**Age (years)**	0.544 ± 0.037	(0.473–0.616)	0.226	41	0.467	0.557
**Basal fT3 (pmol/L)**	0.529 ± 0.041	(0.449–0.609)	0.480	11.40	0.707	0.443
**Thyroid gland volume (ml)**	0.594 ± 0.044	(0.508–0.680)	0.031	17.90	0.706	0.508
**Baseline TRAb measured/Upper Limit**	0.628 ± 0.041	(0.547–0.708)	0.002	5.66	0.534	0.700
