# Supplementary material for: Clinical characteristics of Graves’ disease following COVID-19 infection or vaccination: a multicenter case-control study
Source: Turk J Med Sci. 2025 Nov 11;55(6):1381–93. doi: 10.55730/1300-0144.6096 (PMC12779019; doi:10.55730/1300-0144.6096)
Supplement: Supplementary file 1 [file tjmed-55-06-1381-Supplementary_Table_1.docx]

Supplementary Table-1 The distribution of autoimmune diseases

| COVID group (n=5) | Inact-VAC group (n=3) | mRNA-VAC group (n=12) |
| --- | --- | --- |
| 2 Hashimoto's thyroiditis | 1 Type 1 diabetes mellitus | 1 Type 1 Diabetes Mellitus |
| 1 pernicious anemia | 1Premature ovarian failure | 1 Postpartum thyroiditis |
| 1 Premature ovarian failure | 1 euthyroid Hashimoto's thyroiditis | 6 Hashimoto's thyroiditis |
| 1 Sjogren's disease |  | 1 Addison's disease |
|  |  | 1 Pemphigus vulgaris |
|  |  | 2 Gluten-sensitive enteropathy |
